# Preventing Shoulder-Surfing Attack with the Concept of Concealing the Password Objects' Information

**DOI:** 10.1155/2014/838623

**Published:** 2014-05-27

**Authors:** Peng Foong Ho, Yvonne Hwei-Syn Kam, Mee Chin Wee, Yu Nam Chong, Lip Yee Por

**Affiliations:** ^1^Faculty of Computer Science and Information Technology, University of Malaya, 50603 Lembah Pantai, Kuala Lumpur, Malaysia; ^2^Multimedia University, Jalan Multimedia, 63100 Cyberjaya, Selangor, Malaysia

## Abstract

Traditionally, picture-based password systems employ password objects (pictures/icons/symbols) as input during an authentication session, thus making them vulnerable to “shoulder-surfing” attack because the visual interface by function is easily observed by others. Recent software-based approaches attempt to minimize this threat by requiring users to enter their passwords indirectly by performing certain mental tasks to derive the indirect password, thus concealing the user's actual password. However, weaknesses in the positioning of distracter and password objects introduce usability and security issues. In this paper, a new method, which conceals information about the password objects as much as possible, is proposed. Besides concealing the password objects and the number of password objects, the proposed method allows both password and distracter objects to be used as the challenge set's input. The correctly entered password appears to be random and can only be derived with the knowledge of the full set of password objects. Therefore, it would be difficult for a shoulder-surfing adversary to identify the user's actual password. Simulation results indicate that the correct input object and its location are random for each challenge set, thus preventing frequency of occurrence analysis attack. User study results show that the proposed method is able to prevent shoulder-surfing attack.

## 1. Introduction

Text-based passwords are the most common way to secure access to protected resources [1]. The limitations of text-based passwords are well known [2]. For example, a secure text-based password must be random and formed using a combination of uppercase, lowercase, and special characters. However, secure passwords are hard to remember [3]. As such, users have a tendency to choose weak text-based passwords, which are short and easy to remember. Such an approach weakens the password's strength and makes the password easy to guess by an adversary [1]. To overcome the limitations of text-based passwords, many picture-based passwords have been proposed over the years. Picture-based passwords have been touted as memorable and more secure since they do not contain easily guessable elements [2, 4].

Picture-based password systems have been generally categorized into drawmetrics, locimetrics, and searchmetrics systems [5, 6]. Drawmetrics-based systems require users to draw a previously determined pattern on a canvas in order to log in to the system. Locimetrics-based systems require users to select previously determined points in an image for users in order to access the system. Searchmetrics-based systems, on the other hand, require users to select specific target images as their passwords. A challenge set, which consists of some or all of the password pictures and a set of distracter images, would be shown during the authentication session. The users need to identify their password pictures and select the correct “target” pictures based on their password pictures during the authentication process.

Currently, picture-based password systems suffer from several problems, with one of them being the shoulder-surfing attack [7]. Shoulder surfing has always been a problem with picture-based password systems because of the use of the graphical interface [8]. This is especially true with searchmetrics-based systems because the users are required to select certain images, which are all visible on the screen. Some of the proposed picture-based systems require users to select the entire password during an authentication session. Since the user passwords are not concealed in any way, adversaries would be able to retrieve the passwords by observing a single or multiple login sessions.

In order to secure a picture-based authentication scheme, it is important to conceal information about the password images as much as possible. Over the years, several shoulder-surfing resistant methods have been proposed. While they are able to conceal certain information about the user's passwords, they have drawbacks such as usability and security issues. For example, an adversary would easily retrieve the user's password [9, 10] if multiple sessions are shoulder-surfed. Systems proposed in [10, 11] have reduced password space due to mapping of multiple password images to one input. The polygons formed by the password images in the system proposed by [12] affect usability and security of the system if they are too small or too large.

In this paper, a picture-based password scheme that uses the concept of concealing information about the password images as much as possible is proposed. The password images chosen by the users during the password identification phase are used to form a chain of steps to determine the “target” image in the challenge set. This scheme serves to reduce the amount of information that an adversary can retrieve from observing an authentication session compared to other picture-based password approaches.

The remainder of this paper is organized as follows.  Section 2 presents the related work and Section 3 presents the proposed method. Simulation and the user study results are discussed in Sections 4 and 5, respectively. A conclusion is presented at the end of the paper to summarize the deliverables of the proposed method.

## 2. Related Work

There have been many searchmetrics-based systems proposed over the years. One of the earliest systems is Passfaces [13], introduced in 2000, which requires users to select four pictures of human faces as their password. During the authentication process, users are presented with a challenge set that consists of one password face image together with eight distracter images. Users are required to select their password images in four sequential attempts during the login phase. Studies on the system show that while it was easier for the users to remember their password, it suffers from limited password space, users are having the tendency to select certain pictures, and people with face blindness are unable to use the system [14]. The system is also vulnerable to shoulder surfing as an adversary can easily obtain the password by observing the authentication session. Davis et al. [14] created a similar system with Passfaces but replaced human face images with nonhuman face images and encouraged users to create a story to connect the images together to help them memorize their password images. However, like the Passfaces system, it, too, is vulnerable to shoulder-surfing attack.

The Déjà Vu system, proposed by Dhamija and Perrig [15] in the year 2000, takes advantage of the human ability to remember images even if seen for a short duration of time. It uses random art images, which are hard to describe to reduce the likelihood of users writing down their password images or telling it to another person. Users are presented with a grid during the authentication session where they have to choose their password images among distracter images during individual login attempts. Similar to the Passfaces system, the Déjà Vu system is vulnerable to shoulder-surfing attack as the users select their password images for each of the authentication sessions. Guessing attack is also possible if the adversary knows the user well [16–18].

Visual identification protocol (VIP) was proposed by De-Angeli et al. in 2003 [19]. The authors proposed three different configurations. In the first and second configurations, users select four pictures as their password, which appears during the authentication among five distracter images. Users are required to select their password images in the authentication process. The difference between the first and second configurations (VIP1 and VIP2) is that, in the VIP2 configuration, the locations of the pictures are randomized at the beginning of an authentication attempt, whereas, in the VIP1 configuration, the locations of the pictures are always the same. In the third configuration (VIP3), users select eight pictures of which four will be selected randomly to appear in the challenge set that consists of 16 images in a 4 × 4 grid. Users are required to select the password from images that appear in the challenge set. Por, in 2013, extended the VIP protocol [20], by making certain distracter images appear more often than the other distracter images, which serve to confuse an adversary into thinking that a distracter image is one of the password images. Both VIP1 and VIP2 are vulnerable to shoulder surfing and the adversary would be able to retrieve the password from observing a single login attempt. Both the VIP3 configuration and Por's extended VIP3 are able to reduce the risk of shoulder surfing because only part of the password is revealed in a single authentication session. However, each session reveals a portion of the password. When multiple authentication sessions are observed, the password would eventually be revealed.

In 2004, Roth et al. proposed a PIN input system that conceals the PIN through indirect input [10]. Half of the numbers in the PIN input panel are colored black, while the other half are colored white. Users are then required to select the color that corresponds to the PIN number. The PIN numbers are not directly selected in the process; thus an adversary would be unable to retrieve the users' password. This enables the system to be shoulder-surfing resistant. However, the possible inputs of the system are reduced to either black or white, which increases the vulnerability of the system to guessing attack. In addition, by observing multiple login sessions, an adversary would be able to find out the users' passwords easily by ruling out numbers that do not map to the user input.

Wiedenbeck et al. [12] introduced the convex hull click (CHC) system in 2006. During the authentication session, users are presented with a panel where images are randomly positioned. Three to five of the images shown would be the user's password images. Users have to find these images, mentally form a polygon from them, and click anywhere within the polygon. Multiple rounds are needed for the users in order to log in to the system. This method uses the area within the convex hull as the “target” password to log in. Thus, such a method allows the system to conceal the amount of password images used in the challenge set from the adversary as the users only click once for each challenge set. However, this method has several drawbacks. Since users can form a polygon using any three of the password images, an adversary can use this to his or her advantage while launching an attack as explained in [21]. This method also encounters another problem when a user uses more password images. Using more password images would increase the chance of forming a large polygon and it would allow a large area to be used as the “target” password. Therefore, it increases the vulnerability to guessing attack.

To alleviate the aforementioned problems, this work aims to propose a method that is able to conceal the user passwords and also the number of password images used. The proposed method is also able to prevent someone that has knowledge of a subset of the password images from logging in. In addition, increasing the amount of password images does not weaken the password strength. The following section explains the proposed method in detail.

## 3. Proposed Method

**Figure 1:**
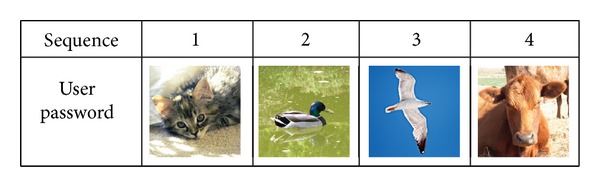
An example of user password pictures and its sequence.

There are two phases in the proposed method: Registration Phase and Authentication Phase. During the Registration Phase, users are required to select multiple images as his or her password. Users are also required to remember the sequence in which the password images were selected. During the password selection process, an image can only be used once. Duplication of images in the password selection is not allowed because it decreases the randomness of the proposed system. An example of the user password images and its sequence is shown in Figure 1.

**Figure 2:**
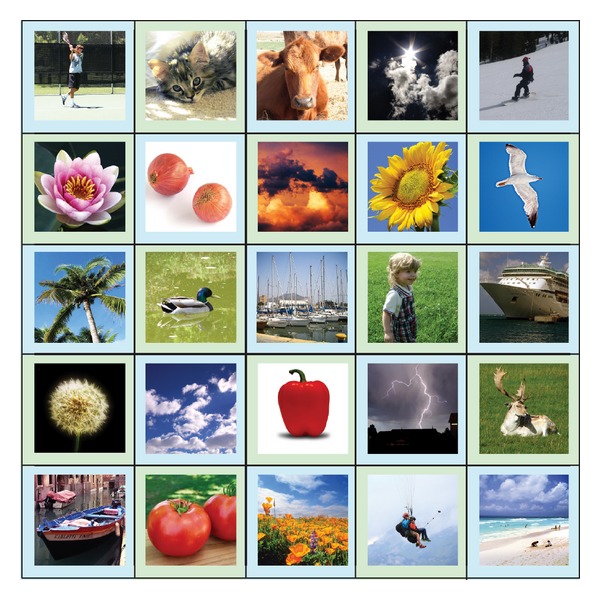
Login screen.

**Figure 3:**
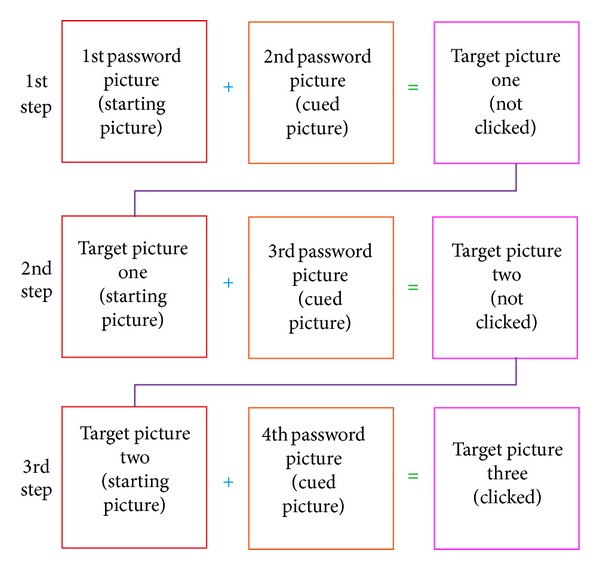
Relationship between starting, cued, and “target” pictures.

During the Authentication Phase, a user is required to identify the correct “target” images within a grid of *N* × *N* images, where *N* is the grid size (refer to Figure 2). The pictures shown in the grid are randomly shuffled. The users are required to click on the final “target” picture in the grid with the aid of the sequence of the registered password pictures. In order to log in, the user has to mentally go through a series of steps. For each step, there will be a starting picture, a cued picture, and a target picture, denoted as *P*_start_, *P*_cued_, and *P*_target_, respectively. Initially, the starting picture and the cued picture can be determined using the first and second password pictures (registered by the user during the Registration Phase), respectively. From both the starting and cued pictures, the user has to identify the first “target” picture using the proposed algorithm. The first step is completed once the first “target” picture is determined (refer to Figure 3). In the next step, the user has to find the next “target” picture with the aid of the current starting and cued pictures. In this step, the first “target” picture is used as the current starting picture and the current cued picture is obtained from the next registered password picture (third password picture). The same algorithm is used to determine the next “target” picture. This process is repeated until the last step when the final “target” picture is obtained. The user is required to click on the final “target” picture and a challenge round is considered complete. The number of steps in the Authentication Phase is always one less than the number of password images selected.

The number of challenge rounds is arbitrary and can be increased to suit the level of security required, thus improving the password space, though the amount of mental work expended by the user increases accordingly.

During each step described above, a starting picture and a cued picture are used to produce a “target” picture. This is achieved by applying an offset to the starting picture. There are many ways that can be used to determine the offset that is to be applied to the starting picture. However, for an authentication method to be secure, the final “target” picture or the output of the method should appear to be randomly selected. If the combination of rules introduces a disproportionately high probability of the users selecting certain pictures or certain location (e.g., center of the challenge set's grid), the output of the algorithm is predictable and insecure.

For the final “target” picture to appear random, any picture on the challenge set should have a chance of being selected as the final “target” picture. This means that no matter where the first starting picture is the final “target” picture can be anywhere in the challenge set's grid. To achieve this, rules of how cued pictures are used should be able to sometimes produce a large offset and, at other times, small offsets. Larger offsets ensure that the entire grid in particular the edges can be “reached,” while smaller offsets ensure that the final “target” picture location is not always near the edges of the grid.

In order to calculate the offset applied to the starting picture, the users have to determine the following criteria:

1. direction of the offset to be applied;
2. amount or magnitude of the offset.

**Figure 4:**
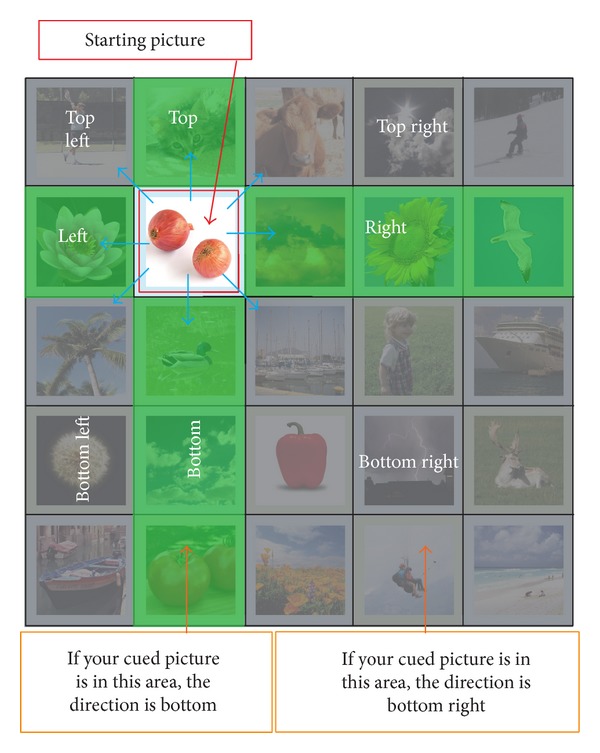
Directions obtained from the cued picture.

**Figure 5:**
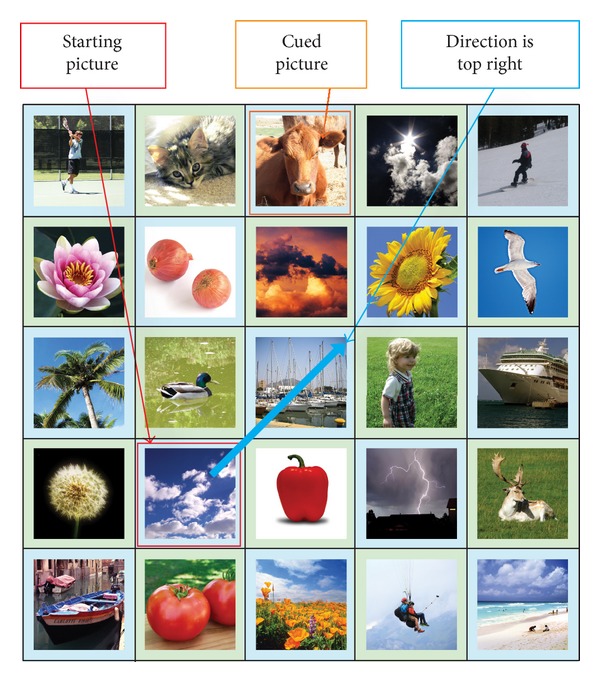
An example in determining direction.

The direction of the offset is the direction of the cued picture, located at (*x*_cued_, *y*_cued_) from the starting picture, located at (*x*_start_, *y*_start_). The direction can be one of the eight directions as shown in Figure 4. An example of how to determine the direction is shown in Figure 5. If the cued picture is the picture highlighted by the orange rectangle, the direction would be top right, indicated by the blue arrow.

The direction of the cued picture consists of two components (*x*- and *y*-axes). The components can be determined by (1). Let *K*_*x*_ and *K*_*y*_ denote the *x* and *y* axes of the direction, respectively. *K*_*x*_ and *K*_*y*_ are defined as

(1) $$\begin{array}{c} K_{x}=\{\begin{array}{cc} 0, & \text{if}\;x_{\text{cued}}-x_{\text{start}}=0,\\ \frac{x_{\text{cued}}-x_{\text{start}}}{\left|x_{\text{cued}}-x_{\text{start}}\right|}, & \text{if}\;x_{\text{cued}}-x_{\text{start}}\neq 0, \end{array}\\ K_{y}=\{\begin{array}{cc} 0, & \text{if}\;y_{\text{cued}}-y_{\text{start}}=0,\\ \frac{y_{\text{cued}}-y_{\text{start}}}{\left|y_{\text{cued}}-y_{\text{start}}\right|}, & \text{if}\;y_{\text{cued}}-y_{\text{start}}\neq 0. \end{array} \end{array}$$

**Figure 6:**
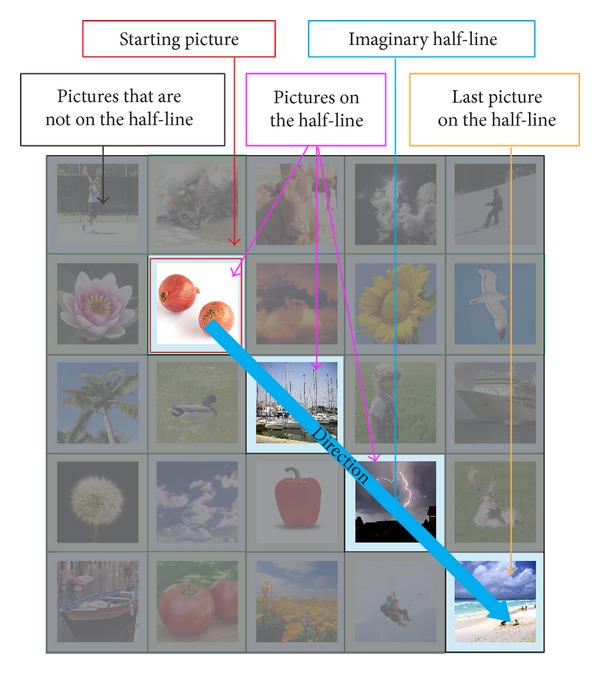
Determining whether a cued picture is on the half-line.

After determining the direction of the offset, the users are then required to find out the amount of offsets. To do this, users are required to imagine a half-line from the starting picture using the determined direction. The users are then required to determine whether their cued picture is on the imaginary half-line. An example illustrating this concept is shown in Figure 6. There are 2 possible scenarios that can occur:

1. cued picture is not on the imaginary half-line;
2. cued picture is on the imaginary half-line.

The two scenarios would result in different target pictures.

**Figure 7:**
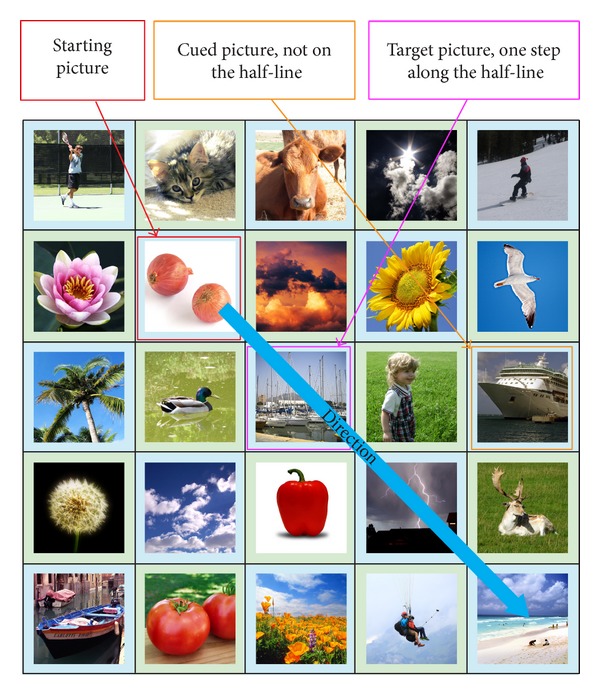
Scenario 1: cued picture is not on the half-line.

The first scenario happens when the cued picture is not on the imaginary half-line. In this case, the amount of offset is fixed to one (refer to Figure 7), which is a small amount of offset. This offset amount was chosen to cater for cases where the starting picture is an immediate neighbor of a picture that is on the edge of the grid. As the starting picture is only “one picture” away from being at the edge, an offset larger than one (in the direction of the edge) would mean going beyond the borders of the grid, which is not allowed.

**Figure 8:**
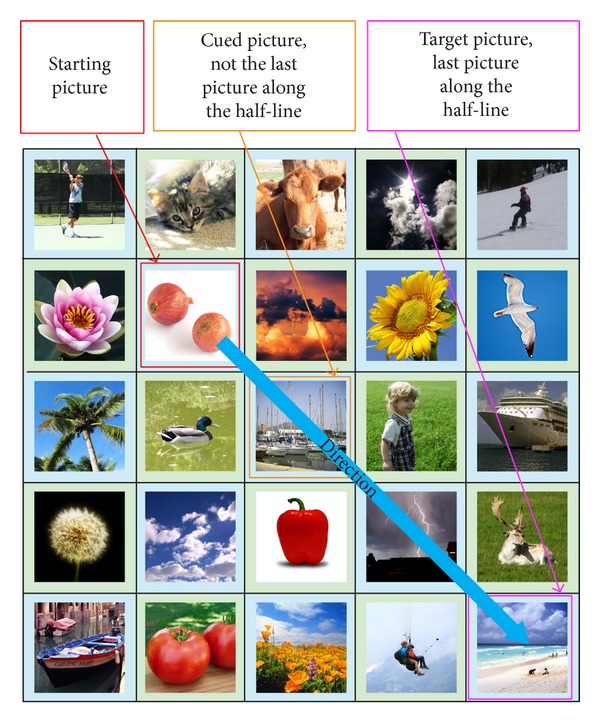
Scenario 2: cued picture on the half-line and not the last picture.

**Figure 9:**
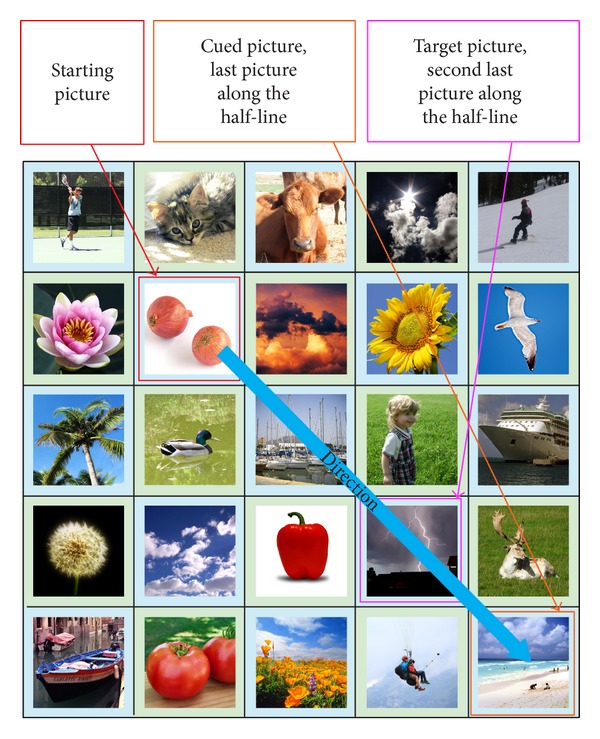
Scenario 2's exception: cued picture is on the half-line and is the last picture.

For the second scenario, the cued picture is on the imaginary half-line. The “target” picture is the last picture along the imaginary half-line (refer to Figure 8). This means “move” to the end of the imaginary half-line to get to the “target” picture. An exception happens when the cued picture is also the last picture on the imaginary half-line, where the offset amount is reduced by 1. In this exception case, the second last picture on the imaginary half-line (refer to Figure 9) is the “target” picture. The reason for this exception is because the password pictures have a much higher chance compared to distracter pictures to be selected as the final “target” picture. A simulation was conducted using all possible ways that four password images can be arranged in a 5 × 5 grid and the final “target” picture was recorded. Without the exception, password pictures and distracter pictures have an average of 9.00% and 3.00% chance of being selected as the final “target” pictures, respectively. With the exception added, the values changed to 4.33% and 3.94%, respectively. This means that, with the exception added, the final “target” picture will be more random (the ideal random value is 4.00% when using a 5 × 5 grid) instead of being one of the password images most of the time. This second scenario would result in an offset amount that ranges from 0 to (*N* − 1) in an *N* × *N* grid, where *N* is the grid size. This means that this scenario would have the following characteristics:

1. producing an offset amount that is not fixed and only known to the user;
2. having a chance of producing large offset amounts. As mentioned above a mix of large and small offsets allow all pictures in the grid to be “reached.”

**Figure 10:**
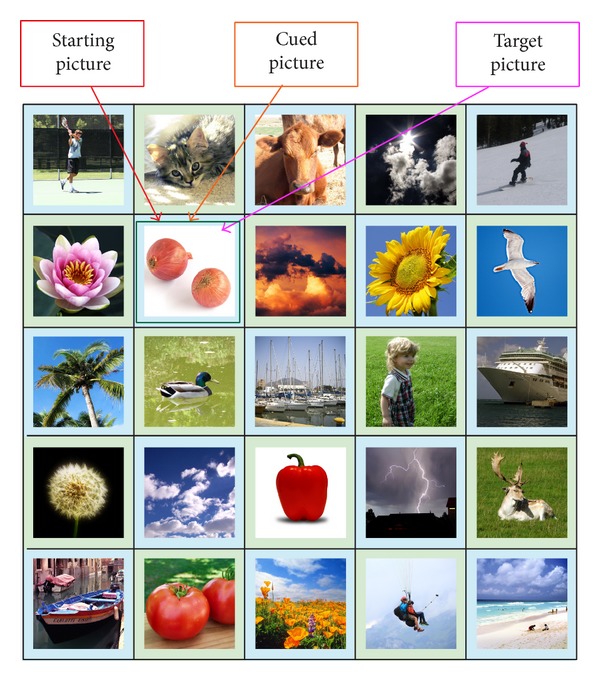
A special scenario: starting picture is the same with cued picture.

Sometimes, after a step is performed and a “target” picture is obtained, the “target” picture, which will be used as the next starting picture, is coincidentally the next cued picture. In this special scenario, the starting picture, the cued picture, and the “target” picture are effectively the same picture (refer to Figure 10). In this case, no offset will be applied. The “target” picture will be the same as the starting or cued picture. The special scenario allows the cued picture to be selected as the “target” picture. This addition allows the algorithm to be more random as the combination of the different scenarios allows the target picture to be the starting picture, cued picture, or other pictures.

The offset amount of the proposed algorithm can be determined by (2)–(4). Let *J*_*x*_ and *J*_*y*_ denote the distance to the location at the end of the imaginary half-line from *P*_start_. *J*_*x*_ and *J*_*y*_ are defined as

(2) $$\begin{array}{c} J_{x}=\{\begin{array}{cc} \begin{array}{c} 0,\\ x_{\text{start}},\\ N-1-x_{\text{start},} \end{array} & \begin{array}{c} \text{if}\;x_{\text{cued}}-x_{\text{start}}=0,\\ \text{if}\;x_{\text{cued}}-x_{\text{start}}<0,\\ \text{if}\;x_{\text{cued}}-x_{\text{start}}>0, \end{array} \end{array}\\ J_{y}=\{\begin{array}{cc} \begin{array}{c} 0,\\ y_{\text{start}},\\ N-1-y_{\text{start},} \end{array} & \begin{array}{c} \text{if}\;y_{\text{cued}}-y_{\text{start}}=0,\\ \text{if}\;y_{\text{cued}}-y_{\text{start}}<0,\\ \text{if}\;y_{\text{cued}}-y_{\text{start}}>0. \end{array} \end{array} \end{array}$$

Let *L*_*x*_ and *L*_*y*_ denote the offset amount on *x* and *y* axes, respectively, prior to the adjustment for scenario 2's exception. *L*_*x*_ and *L*_*y*_ are defined as

(3) $$\begin{array}{c} L_{x}=\{\begin{array}{cc} 1, & \text{if}\;\left(y_{\text{cued}}-y_{\text{start}}\neq 0\right),\\ & \;\left(\left|x_{\text{cued}}-x_{\text{start}}\right|\neq \left|y_{\text{cued}}-y_{\text{start}}\right|\right),\\ J_{y}, & \text{if}\;\left(x_{\text{cued}}-x_{\text{start}}\neq 0\right),\\ & \;\left(\left|x_{\text{cued}}-x_{\text{start}}\right|=\left|y_{\text{cued}}-y_{\text{start}}\right|\right),\\ & \;\left(J_{x}>J_{y}\right),\\ J_{x}, & \text{otherwise}, \end{array}\\ L_{y}=\{\begin{array}{cc} 1, & \text{if}\;\left(x_{\text{cued}}-x_{\text{start}}\neq 0\right),\\ & \;\left(\left|x_{\text{cued}}-x_{\text{start}}\right|\neq \left|y_{\text{cued}}-y_{\text{start}}\right|\right),\\ J_{x}, & \text{if}\;\left(y_{\text{cued}}-y_{\text{start}}\neq 0\right),\\ & \;\left(\left|x_{\text{cued}}-x_{\text{start}}\right|=\left|y_{\text{cued}}-y_{\text{start}}\right|\right),\\ & \;\left(J_{y}>J_{x}\right),\\ J_{y}, & \text{otherwise}. \end{array} \end{array}$$

The offset amounts |Offset(*P*_start_, *P*_cued_)|, adjusted for the scenario 2's exception, are denoted as *M*_*x*_ and *M*_*y*_ for *x* and *y* axes, respectively. They are defined as

(4) $$\begin{array}{c} M_{x}=\{\begin{array}{cc} L_{x}-1, & \text{if}\;\left(L_{x}\neq 0\right),\\ & \;\left(x_{\text{start}}+K_{x}L_{x}=x_{\text{cued}}\right),\\ & \;\left(y_{\text{start}}+K_{y}L_{y}=y_{\text{cued}}\right),\\ L_{x}, & \text{otherwise}, \end{array}\\ M_{y}=\{\begin{array}{cc} L_{y}-1, & \text{if}\;\left(L_{y}\neq 0\right),\\ & \;\left(x_{\text{start}}+K_{x}L_{x}=x_{\text{cued}}\right),\\ & \;\left(y_{\text{start}}+K_{y}L_{y}=y_{\text{cued}}\right),\\ L_{y}, & \text{otherwise}. \end{array} \end{array}$$

Finally, the location of the “target” picture is defined as

(5) $$\begin{array}{c} P_{\text{target}}=\left(x_{\text{start}}+K_{x}M_{x},y_{\text{start}}+K_{y}M_{y}\right). \end{array}$$

**Figure 11:**
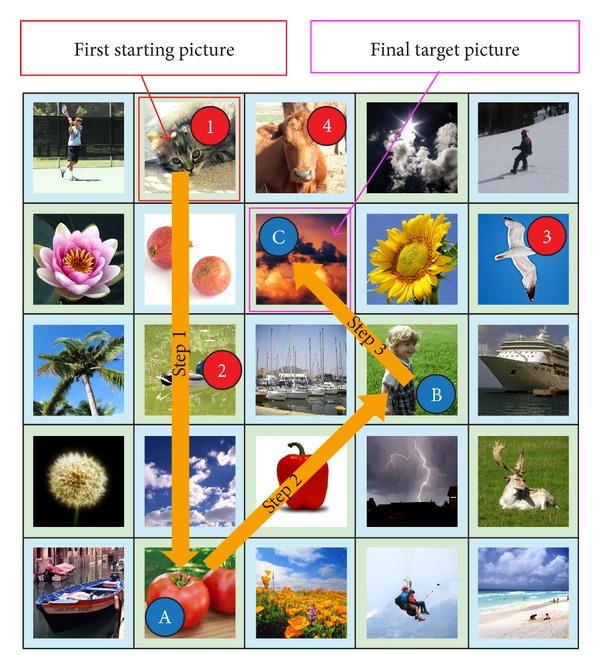
An example of a challenge round.

An example of a single challenge round is shown in Figure 11. The password pictures used are marked with numbers from 1 to 4, while the “target” pictures for steps 1, 2, and 3 are marked with A, B, and C, respectively. For the first step, the direction is “bottom” and the cued picture (password picture 2) is on the imaginary half-line and is not the last picture on the imaginary half-line. For the second step, the direction is “top right” and the cued picture (password picture 3) is on the imaginary half-line and it is also the last picture on the imaginary half-line. Hence, the “target” picture in this step is picture B. For the third and final step, the cued picture (password picture 4) is not on the imaginary half-line. Thus the final “target” picture is picture C. The users are required to select the final “target” picture (picture C) to complete the challenge round. The pseudocode of the proposed algorithm is shown in Algorithm 1. 

The final “target” picture is the combination from all the offsets applied in each of the steps. The combination of offsets allows the final “target” picture to be any picture in the grid. When the password pictures are placed in a different order, one or more of these will also change

1. first starting picture's location;
2. offsets to be applied in each step.

The correct user input (final “target” picture) changes when there are changes in the above-mentioned items. However, the user input does not reveal the password pictures to a shoulder-surfing adversary. This means that the password pictures are concealed from shoulder-surfing adversaries. Sometimes, the proposed method would result in password pictures as the final “target” pictures. Because of this, information of whether the user selected picture is a user password picture or a distracter picture is concealed. This further conceals password information from any adversary.

## 4. Simulation

A simulation was performed using all possible ways that the four password pictures can be ordered in a 5 × 5 grid. The purpose of this simulation is to test the randomness and the vulnerability of the proposed method toward frequency of occurrence analysis attack mentioned by [20]. Ideally, the probability of any picture being selected as the final “target” picture is based on uniform randomization. If the final “target” picture always happens to be a password picture, one can simply observe a login session and repeat the picture selection that the user made. In other words, it would mean that the proposed system is vulnerable to frequency of occurrence analysis attack [20].

Table 1 shows the simulation result with the percentages of final “target” pictures that are also password pictures for the proposed system. The uniform randomization value of 4% is indicated as the ideal value and is used as a comparison with the percentage of final “target” pictures that happen to be a particular password picture. From the table, the percentage of the first, second, third, and fourth password pictures that becomes the final “target” picture is 5.40%, 3.69%, 4.43%, and 3.78%, respectively. On average, the overall result is very close to the ideal value. This means that the final “target” picture selected using the proposed algorithm is very close to random and it does not bias towards the selection of the password picture to become the final “target” picture. This also means that a shoulder-surfing adversary cannot determine whether the picture clicked by a user is a password picture or distracter picture. Launching a frequency of occurrence analysis attack against the proposed method will not be successful.

**Figure 12:**
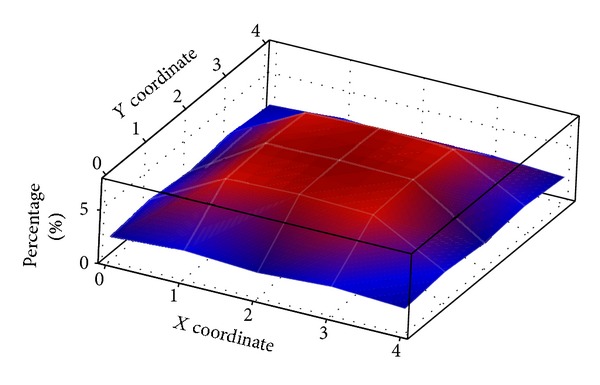
Distribution of the final “target” picture using the proposed method.

The distribution of the final “target” picture locations across a 5 × 5 grid is plotted using a heat map as shown in Figure 12. The *X* and *Y* axes represent the *x* and *y* coordinates of the grid cell, respectively, while the *z*-axis represents the occurrence of the final “target” picture in percentage of times. Higher values are indicated with warm colors (hues from red through yellow) and cool colors (hues from blue and green through blue violet) are used to represent lower values. The purpose of conducting this testing is to investigate whether the proposed method is location dependent. If the final “target” picture is always located at a single location (e.g., center of grid), it might become a potential threat. According to the map, the final “target” pictures generated by the proposed algorithm are distributed to all the locations. On average, the tendency for the final “target” picture to be located at the edges of the grid is 41.0% (blue areas in the map) compared to the other parts of the grid (59.0% is covered by red areas). There is no outstanding point shown in the graph. Thus, launching a location detection analysis attack towards the proposed method will be to no avail.

## 5. User Study

### 5.1. Participants

A user study was conducted to test the effectiveness of the proposed method in reducing shoulder-surfing attack. We sent out email invitations for volunteers to try out the proposed method. Thirty people responded; 19 of them were male, while 11 of them were female. All of them are computer literate. The participants were not compensated for their participation in the user study.

### 5.2. Procedure

A web-based application was created and invitations were sent to the participants. A web-based tutorial was made to show the participants the concept of the system and the steps that they should use in order to log in. The participants were told to first undergo the tutorial before creating their password and logging in to the system.

A 5 × 5 grid of images was used as the login screen in this study. In the Registration Phase, the participants were required to select four pictures as their password images. They were instructed to use the password images for ten successful login attempts to familiarize them with the method. Each login attempt requires users to go through three challenge rounds. The time used to log in and the frequency of errors were recorded by the system. After the participants had completed ten successful logins, they were instructed to watch a video of a login session. The resources available to them as the attackers are the images shown in the challenge set and the images clicked by the authorized user during login. Then, they were given unlimited trials to guess the password pictures used for the login session in the video. After that, they were given a posttest questionnaire to find out the shoulder-surfing strategy that they used and their feedback was collected. Each of the participants was tested separately.

### 5.3. Results

Each of the participants completed ten successful logins (3 challenge rounds were required for each login attempt). 14 of the participants of the user study did not make any incorrect login attempts during the user study, whereas the remaining 16 participants made 1 to 5 incorrect login attempts. The average probability of a successful login attempt is 86.21% based on the pool of participants in the survey.

**Figure 13:**
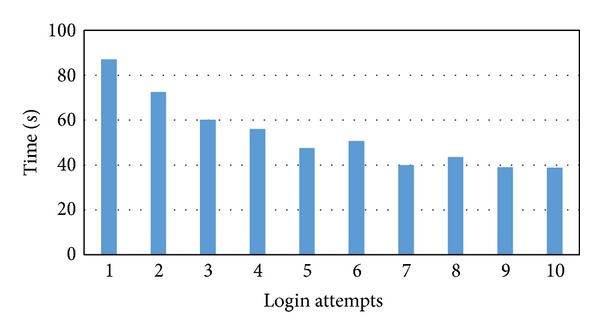
Mean times in seconds for 10 successful logins.

Figure 13 shows the mean times of ten successful login attempts. Each login attempt consists of three challenge rounds. The chart indicates that, over the ten successful login attempts, the login time for the participants decreased significantly as the participants gained more experience with the system.

Table 2 shows the minimum, maximum, average, and standard deviations for all successful login attempts. The minimum time taken by the participants for a successful login attempt was 16.1 seconds, while the maximum time taken was 184.2 seconds. The participants took an average of 53.5 seconds to log in to the systems, with the standard deviation being 33.1. The median for all the successful login attempts is 42.9 seconds. This indicates that 50% of the successful login attempts require less than 43 seconds to log in.

The shoulder-surfing test resulted in none of the respondents being able to identify the password images used in the video even though they were given unlimited trials to guess the password used with the help of a video recorded login session. One would suspect that if the adversary knows the algorithm used, it might give them an advantage to accomplish their attacks. The results of the shoulder-surfing test in the user study suggest that the proposed system is resistant to shoulder-surfing attacks (both traditional and video recorded shoulder-surfing attacks), despite the fact that the attackers know how the proposed system and the underlying algorithm work.

The participants were given a posttest questionnaire and were asked about the strategy they used to obtain the password. 43.3% of the participants used a direct observation method such as repeating the selected pictures based on the recorded video, while 13.3% of the participants used simple and commonsense shoulder-surfing strategies such as identifying the location of the selected pictures based on the recorded video. Some other participants (43.3%) just randomly selected some pictures in the challenge set because they did not have any clue as to which pictures are the starting picture, cued picture, and target picture.

## 6. Discussion

The weakness of having limited password spaces is a known disadvantage for searchmetrics-based systems [2, 22]. An adversary can launch a brute-forcing attack by using data from multiple sessions to deduce the password images. There are several methods to reduce the effectiveness of brute-forcing attacks. One of the methods is to increase the password spaces of a proposed system. Increasing the password spaces might render such attacks infeasible due to the amount of memory required to record a significant portion of all possible passwords [12]. In this paper, we focused on preventing shoulder-surfing attacks. In our proposed method, one needs to have knowledge of the full set of password images in order to get the final target image. However, an adversary without this knowledge could launch a guessing attack to guess the password used. To increase the password space, the proposed system can be scaled up (from 5-by-5, with 4 password images) to n-by-n, with more than 4 password images to suit the level of security needed. However, the size of the matrix could be limited by the size of the window/screen and the difficulty to locate password images in a larger grid. Passwords are expected to comply with two fundamentally contradictory requirements; they must be easy to memorize and yet have to be secure. Therefore, a usability study of the authentication process using larger spaces could be carried out [23]. However, such a study is not within the scope of this current research work.

The proposed method uses images as the password, which are said to be easy to remember [2–4]. Works undertaken over the years have also reported that users can remember picture passwords better than alphanumeric passwords [13, 24]. Methods applied in [14, 25], where users are asked to create a story to link the sequence of password images together, can also be used to improve the memorability of the proposed method.

Humans are said to be the “weakest link” in the security chain and unsafe user behaviors often compromise the security of an authentication system [3]. Similarly, the security of a proposed method can be affected by unsafe user behavior. For example, a user might not follow good password practices such as writing down his password and sharing his password with another person [26]. In addition, the user might point at his password images during the login sessions or hover over the starting and target images whilst performing the steps. These behaviors would jeopardize the security of the system. As with any security policy, users need to be advised to follow good password practices to reduce the occurrence of indirect attacks.

## 7. Conclusion

In this paper, a new searchmetrics picture-based password authentication method that is resistant to shoulder-surfing attack was proposed. The proposed method uses a concept of concealing password information as much as possible from prying eyes. On top of concealing the password images (which gives the proposed method its shoulder-surfing resistant properties) and the amount of password images, the proposed method also conceals whether the user input is a password picture or a distracter picture. A simulation was conducted to find out the final “target” pictures that are also password pictures and the distribution of these final “target” pictures in a 5 × 5 grid. The result of the simulation shows that the final “target” picture selected using the proposed algorithm is random and it does not bias towards the selection of a password picture to become the final “target” picture. Moreover, the final “target” pictures generated by the proposed algorithm are distributed across all the grid locations. This means that the proposed method is able to resist the frequency of occurrence analysis and location detection analysis attacks. A user study was conducted. The result shows that the proposed method is able to resist shoulder-surfing attack.

In the future, research into other graphical authentication methods such as the use of fake movement to mislead attackers as in [27] and improving other security aspects like increasing the password length and usability issues such as helping the users to memorize their password will be our main focus. In addition, because of the indirect method of selecting pictures, password hashing is not feasible. This allows someone who has access to the database to view the users' passwords. To overcome this, data hiding methods detailed in [28–32] will be considered to secure the registered password for strengthening the proposed system.

## Figures and Tables

**Algorithm 1:**
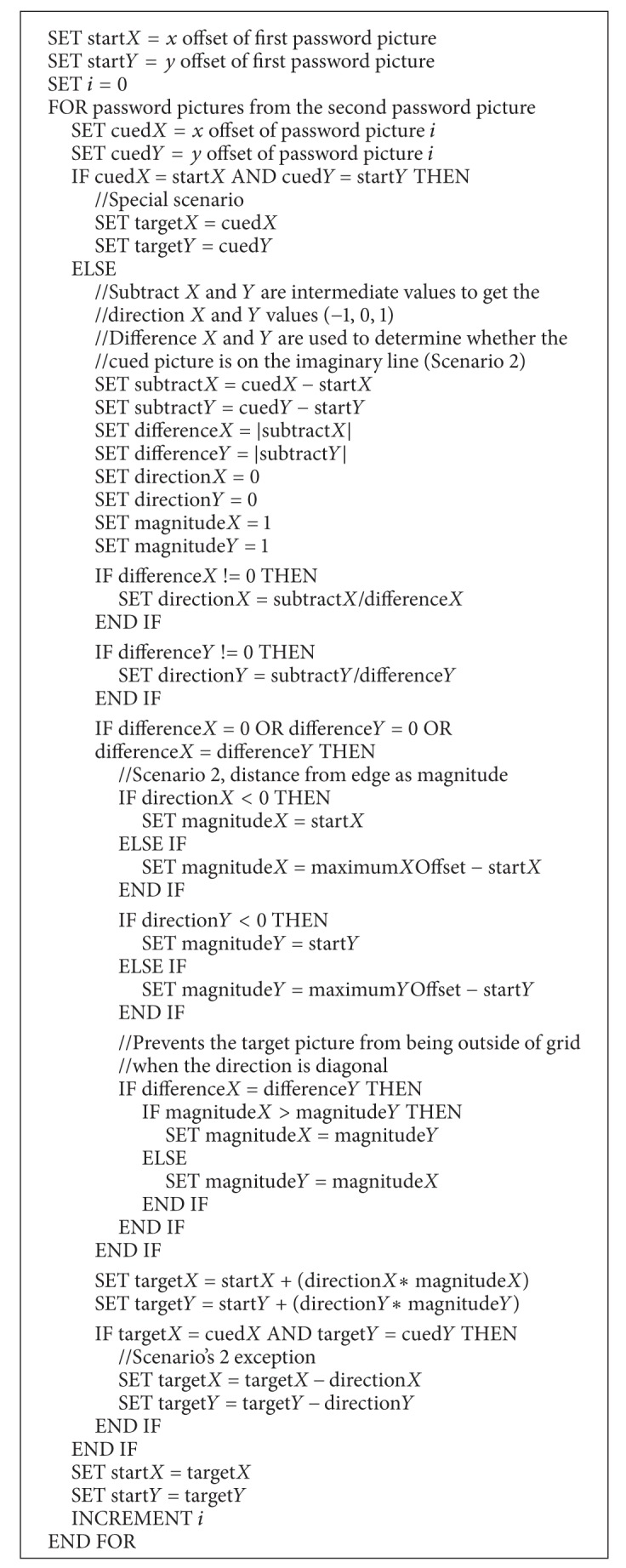
Pseudocode for the proposed algorithm.

**Table 1:** Percentages of final “target” pictures that are also password pictures.

Password picture	1st	2nd	3rd	4th	Ideal
Percentage of final “target” pictures that are also password images	5.40	3.69	4.43	3.78	4.00

**Table 2:** Statistics of successful login time.

Item	Time (seconds)
Minimum	16.1
Maximum	184.2
Average	53.5
Standard deviation	33.0
Median	42.9
